# Effects of Raw and Pasteurized Camel Milk on Metabolic Responses in Pigs Fed a High-Fat Diet

**DOI:** 10.3390/ani12131701

**Published:** 2022-06-30

**Authors:** Kristy DiGiacomo, Fernanda Zamuner, Yushu Sun, Frank R. Dunshea, Jared K. Raynes, Brian J. Leury

**Affiliations:** 1 Faculty of Veterinary and Agricultural Sciences, School of Agriculture and Food, University of Melbourne, Parkville, VIC 2010, Australia; fernanda.z@unimelb.edu.au (F.Z.); yushus2@student.unimelb.edu.au (Y.S.); fdunshea@unimelb.edu.au (F.R.D.); brianjl@unimelb.edu.au (B.J.L.); 2 Faculty of Biological Sciences, University of Leeds, Leeds LS2 9JT, UK; 3 Faculty of Engineering, School of Chemical and Biomolecular Engineering, The University of Sydney, Sydney, NSW 2006, Australia; jared.raynes@csiro.au

**Keywords:** camel milk, diabetes, insulin resistance, metabolic syndrome, minimal modelling

## Abstract

**Simple Summary:**

Camel milk (CM) contains insulin-like peptides and is high in vitamin C, vitamin E, and antioxidants. Previous studies in diabetic mice and humans have demonstrated a positive impact of CM consumption on glycemic balance, potentially greater than that observed for the consumption of bovine milk. Thus, CM may be a viable therapeutic treatment for diabetic humans, although the mode of action of these effects are not yet understood. This experiment used a high-fat diet as a monogastric model to examine the effect of CM consumption (raw or pasteurized) on some key blood metabolic markers and examined responses to an in vitro glucose tolerance test. While the results are preliminary given the low number of animals, this experiment suggested that CM can improve glycemic control, potentially via a tighter control of insulin effectiveness and/or uptake.

**Abstract:**

Evidence suggests that camel milk (CM) can have insulin-like actions, although the mode of action is not understood. Using the pig as a monogastric model, this pilot experiment examined the effects of CM consumption on metabolic responses to an in vitro glucose tolerance test (IVGTT). Twenty female Large White × Landrace pigs were individually housed for 6 wks and randomly allocated to one of the following four diets (fed ad libitum; *n* = 5): control (Con); high fat (HF; ~16% fat); raw CM (the HF diet plus 500 mL CM/ day); or pasteurized CM (PCM). Blood samples were collected on two occasions (weeks 2 and 5). At week 6, the pigs were fitted with an ear vein cannula and the following day an in vitro glucose tolerance test (IVGTT) was conducted (0.3 g/kg BW glucose). Plasma fatty acids and cholesterol concentrations were greater in the pigs fed the HF diet and greatest in those fed CM, while there was no effect of diet on insulin concentrations. The pigs fed CM tended to have a reduced peak insulin (*p* = 0.058) and an increased glucose nadir (*p* = 0.009) in response to the IVGTT. These preliminary results tend to support the hypothesis that feeding CM can improve glycemic control in pigs.

## 1. Introduction

Camel milk (CM) contains insulin and insulin-like peptides that have biological and pharmacological properties much greater than that noted in bovine milk and is high in vitamin C, vitamin E, glutathione, and other antioxidants [1]. The concentration of insulin peptides in CM is three-fold greater than in bovine milk (58.7 IU/L vs. 17.0 IU/L) [2]; this peptide is also more resistant to gastric degradation and thus elicits a larger hypoglycemic action as more is absorbed via the small intestine. These peptides are also processed via the liver, meaning it can mimic the effects of pancreas-secreted insulin by inhibiting hepatic gluconeogenesis [3]. Thus, CM has been explored as a potential treatment for both Type 1 and Type 2 diabetes. Recent evidence in Type 1 diabetic rat models demonstrated that CM had no impact on body weight (BW) or basal plasma glucose concentrations, while in response to a glucose tolerance test (GTT) CM fed diabetic rats showed decreased blood glucose and improved glucose tolerance compared to control. In addition, raw CM reduced (−210%) fasting circulating glucose concentrations in Type 1 diabetic rats over a three-week period [4]. A systemic review by Mirmiran et al. (2017) concluded that most published studies demonstrated the favorable effects of camel milk on diabetes mellitus by reducing blood sugar, decreasing insulin resistance, and improving lipid profiles [5]. Thus, CM has been promoted as a therapeutic alternative to drugs for Type 1 and Type 2 diabetes mellitus patients [6]. The use of CM to treat diabetes is particularly promising as it can be delivered orally and therefore does not require the pain associated with injectable and inconvenient therapeutic treatments.

While there appears to be evidence supporting the ability of CM to act as an insulin replacement and/or improve de novo insulin production, much of this information is not published in peer reviewed journals, not supported by controlled studies, or is conducted in rodents or species that are not directly comparable to humans. To our knowledge, only one published study has measured responses to a glucose tolerance test in CM-fed animals, which was conducted in rats [7]. The pig is an ideal animal model for such studies as they are the closest relevant animal model for comparison to humans in terms of digestive and metabolic functions [8,9,10]. This experiment will therefore use pigs as an animal model for Type 2 diabetes mellitus by inducing a mild form of metabolic syndrome (a degree of insulin resistance, i.e., pre-diabetes) through a high fat diet to subsequently allow the incorporation of diets containing either raw or pasteurized CM to measure the insulinogenic effects of dietary CM. Raw and pasteurized milks were compared as heat treatment may potentially eliminate the favorable insulinogenic effects of CM.

## 2. Materials and Methods

All animal procedures were approved by the University of Melbourne Faculty of Veterinary and Agricultural Sciences Animal Ethics Committee (approval number 1914753.1). Twenty female Large White × Landrace grower pigs (approx. 35 kg initial liveweight) were selected from a commercial piggery (Berrybank Farm, Windamere, Victoria) and transported to the FVAS large animal house (approx. 2.5 h, 220 km) using a covered and well-ventilated stock truck. Upon arrival and throughout the experiment, the pigs were housed in individual pens with constant visual and audial contact with other pigs. After initial arrival in the animal house, pigs were acclimatized to their individual pens and feeding regimes over a 10-day period. During the first 5 days of acclimatization, pigs were fed a standard grower diet (control, Con). Starting from day −6, pigs were gradually introduced to their experimental diets until the full diet was given on days 8–10 of acclimatization. Pigs were randomly allocated to one of four dietary treatments (*n* = 5 per treatment):

1. Low fat diet (control)
2. High fat diet (HF) (the main source of fat was tallow)
3. High fat diet + 500 mL/per day Raw Camel Milk (CM)
4. High fat diet + 500 mL/per day 63 °C Pasteurized Camel Milk (CM)

The experimental diets were fed ad libitum (adjusted daily to allow for approx. 10–15% residual feed each day) twice daily (approx. 0800 and 1600 h) for a period of 6 weeks. Diets were pelleted and formulated to meet or exceed nutrient requirements for growth according to the NRC for swine (2012) [11] (Table 1). Water was available ad libitum via individual nipple drinkers located in each individual pen. Camel milk was sourced from a commercial dairy and delivered chilled throughout the experiment. Sub-samples of each delivery of camel milk during the experiment (*n* = 3 for pasteurized milk and *n* = 5 for raw milk) were obtained and stored at −80 °C prior to analysis. At the end of the experiment, analysis was conducted to measure CM protein, fat, and lactose concentrations. Final data are presented as an average of all sub-samples. There was no difference in the total percentages of protein, fat, and lactose between raw camel milk (3.3, 2.8, and 4.2%, respectively) and pasteurized camel milk (3.3, 2.9, and 4.3%, respectively).

### 2.1. Measurements

#### 2.1.1. Macrocompositional Analysis of CM

CM samples were thawed at room temperature, warmed to 37 °C, and mixed to ensure a homogenous sample. CM samples were then analyzed in triplicate for total protein, fat, and lactose using a PerkinElmer™ LactoScope FT-A.

#### 2.1.2. Liveweight, Backfat, and Feed Intake

Once weekly, pigs were weighed (BW) using standard walk over scales and a weigh crate, and while in the crate backfat was measured at the P2 site (located 65 mm from the edge of the dorsal mid-line at the level of the last rib). After first lubricating the site with electrode gel, a lean-meter (Renco) ultrasound device was used to obtain three consecutive backfat measurements and an average of the three is reported. Daily feed intake was recorded by weighing orts prior to the morning feeding.

#### 2.1.3. Blood Sampling

In weeks 2 and 5 (day 14 and 35), fasting blood samples were taken via external jugular venipuncture prior to the morning feeding. Blood was collected into a 10 mL vacuum tube coated with lithium-heparin (BD Vacutainer, Plymouth, UK), immediately placed on ice, and centrifuged (1250× *g*, 4 °C) for 10 min within 1 h after collection. Isolated plasma was stored at −20 °C until analysis.

#### 2.1.4. In Vitro Glucose Tolerance Test (IVGTT)

On day 43, a catheter was inserted into a marginal ear vein of each pig as per the methods described by [12]. Briefly, pigs were restrained using a snout rope and their ear cleaned with Betadine antiseptic solution and 70% ethanol. A suitably sized and located ear vein was catheterized using an 18 G × 1¼ inch I.V. catheter (Surflo I.V. catheter, Terumo Corporation, Macquarie Park, Australia) and the needle was withdrawn from the catheter. A wire guide with a 0.81 mm diameter and 90 cm length (Radifocus Glidewire, Terumo Corporation, Australia) was passed through the catheter into the vein to a depth of approximately 30 cm. A small incision (1–2 mm) was made using a sterile scalpel at the insertion site and a single lumen polyethylene catheter (0.97 mm ID × 1.27 mm ID, 120 cm in length, Tyco Electronics Pty Ltd., Kingsgrove, Australia) was passed over the wire guide through the incision and into the ear vein to a depth of approximately 35 cm, which placed the catheter tip in an external jugular vein. The wire guide was then removed and the catheter was flushed with heparinized saline (100 IU/mL) and plugged with a sterile sampling port (Safesite). The catheter was secured into a pouch using fabric tape (Elastoplast). Prior to the IVGTT on day 43–44, pigs were fasted for 12 hrs.

Blood samples were collected via the jugular catheter at times −30, −15, and −1 min, then a 0.3 g/kg BW 50% dextrose solution (Baxter Healthcare, Toongabbie, NSW, Australia) was administered intravenously and blood samples were collected at −30, −15, −1, 2, 3, 4, 5, 6, 8, 10, 12, 14, 16, 18, 20, 25, 30, 35, 40, 60, 90, 120, 150, 180, 210, 220, and 240 min from glucose infusion. The catheter was flushed with sterile saline and heparin (diluted to 10 I.U. per ml) between each blood sample collection. All blood samples were collected into a 10 mL vacuum tube coated with lithium-heparin (BD), immediately placed on ice, and centrifuged (1250× *g*, 4 °C) for 10 min within 1 h after collection. Isolated plasma was stored at −20 °C until analysis. At the completion of the challenge, the catheter was flushed with 20 mL sterile saline and heparin (50 IU per ml) and resecured in its pouch.

After the completion of the IVGTT, the ear vein catheter was removed from each pig. On day 45, pigs were transported to a commercial abattoir (Diamond Valley Pork) and slaughtered via standard commercial practices.

### 2.2. Laboratory Analysis

Plasma fatty acids and glucose were analyzed in duplicate (for all plasma samples obtained during the IVGTT) and concentrations were determined spectrophotometrically using commercial kits (NEFA-C (modified as per the methods of Johnson and Peters (1993 [13])), Wako Pure Chemical Industries, Ltd., Osaka, Japan, and Infinity Glucose Oxidase Liquid, Thermo-Scientific, VA, USA, respectively). The inter- and intra-assay coefficients of variation (CV) for glucose were <6.4 and <3.1%, and for fatty acids were <3.5 and <6.3%. Plasma insulin concentration was measured in duplicate (for all plasma samples taken during the IVGTT) using RIA kits (Porcine Insulin Cat. # PI-12K, Millipore Corporation, Billerica, MA, USA), following the manufacturer’s instructions. The insulin intra-assay CV was <10% between 3.6–849.0 µU/L. Plasma urea was analyzed in duplicate for the fortnightly blood samples spectrophotometrically using commercial kits (Infinity Urea reagents, Thermo-Scientific) following the manufacturer’s instructions. The inter- and intra-assay CV for urea were 0.07 and 1.7%, respectively. The plasma total cholesterol, interleukin-10 (IL-10), IL-6, insulin growth factor-1 (IGF-1), IGF-2, glucagon, GLP-1, and C-peptide concentrations were all determined via porcine ELISA kits (Scientifix Pty Ltd., South Yarra, VIC, Australia) as per kit instructions. The inter- and intra-assay (where multiple plates were examined) CVs were (respectively): cholesterol (3.7; NA); IL-10 (2.5; NA); IL-6 (3.3, 6.0); IGF-1 (3.6; NA); IGF-2 (3.3; NA); glucagon (5.9, 9.0); GLP-1 (2.5, 5.0); and C-peptide (4.5; 4.1).

### 2.3. Metabolic Challenges Data Calculations

The baseline concentrations for the measured analytes were calculated as the mean concentration of the 3 blood samples taken prior to the glucose infusion. Plasma hormones and metabolite responses were analyzed for the area under the curve (AUC) using a linear trapezoidal summation between successive pairs of metabolite concentrations after correcting for baseline concentrations. The peak and nadir concentrations, percentage change from baseline, clearance rates (CR), time (T) to reach, peak (Tpeak), recovery (concentration at 240 min), and basal (Tbasal) concentrations were calculated for each pig and mean values are reported for each specific treatment group. Values were calculated using the following formulas, as previously described by Pires et al. (2007) [14].

CR = [(ln [ta] − ln [tb])/(tb-ta)] × 100, where [ta] is the concentration of the metabolite at time a (ta) and [tb] is the concentration of metabolite at time b (tb).
Tbasal glucose = [(ln [2 min] − ln [240 min])/CR2-30 glucose] × 100
Tbasal insulin = [(ln [2 min] − ln [240 min])/CR20-120 insulin] × 100
Increment = peak concentration − basal concentration
Decrement = nadir concentration − basal concentration.
Change = [(peak (or nadir) concentration − basal concentration)/basal concentration] × 100

### 2.4. MINMOD Parameters

Key indices of glucose-insulin dynamics were calculated using MINMOD Millenium software (MINMOD Inc., Pasadena, CA, USA; [15]). The parameters and indices that are output by the MINMOD software are:

GB = basal glucose concentration pre-infusion (mM)
IB = basal insulin concentration pre-infusion (mU/L)
SG = glucose effectiveness (min^−1^), which refers to the capacity of glucose to mediate its own uptake
SI = insulin sensitivity ((mU/L)^−1^.min^−1^), which refers to the capacity of insulin to promote glucose uptake
AIRg = acute insulin response to glucose (mIU/L^−1^.min^−1^), which addresses the adequacy of insulin secretion in response to a glucose bolus
DI = disposition index (AIR × SI), which represents the ability of the islet cells to secrete insulin
G0 = distributed glucose concentration at time 0
GEZI = glucose effectiveness at zero insulin
Other indices that are included in the MINMOD Millennium output are derived from the Homeostatic assessment model (HOMA), as follows:
IR = insulin resistance (mM/mU/L^−2^), calculated by the equation: (IB × GB)/22.5
Β-cell function (BCF) = pancreatic β-cell function (mU/mM), calculated by the equation: (20 × IB)/(GB-3.5)

### 2.5. Statistical Analyses

Statistical analyses were performed using GenStat software (18th edition; VSN international, Hemel Hampstead, UK; [16]). All outcome variables were screened for normality by calculation of kurtosis and skewness and by visual assessment of standardized residuals distribution. When required, data were log transformed. Data from repeated-measurements were analyzed using the ANOVA Mixed Effects Model (restricted maximum likelihood (REML) and 2-sided 95% CI), and data from non-repeated-measurements were analyzed using the ANOVA General linear model (2-sided 95% CI) of Genstat. All models included random effect of pig. Bodyweight was tested in all models as a covariate and was not deemed to be significant (*p* < 0.05) for any model tested and was therefore removed from the model. The Bonferroni post-hoc test with 95% CI was used for pairwise comparisons. Statistical significance was declared at *p* < 0.05, and values of *p* < 0.1 were considered a trend toward significance. Results for log-transformed variables were reported and back-transformed data are shown in parentheses. Data are presented as means ±SE unless declared otherwise.

## 3. Results

### 3.1. Growth, Feed Intake, and Plasma Metabolite Responses

There was no difference in initial (33.6 ± 2.5 kg) or final (86.5 ± 5.8 kg) BW, feed intake, bodyweight gain, or feed conversion efficiency between diet treatment groups. The pigs consuming the HF diet consumed less dry matter (DM) than the control-fed pigs, while there was no impact of CM on feed intake (16.1 vs. 14.6 vs. 14.4 kg/week for Con, HF, and CM, respectively, SED 0.56, *p* = 0.003) driven by the higher energy density of the HF diet. While the pigs grew throughout the experiment, there was no change in backfat due to experimental week or diet (mean 15.1 mm ± 0.51). There was no difference in plasma glucose, fatty acids, or urea concentrations between the measurements obtained at weeks 2 and 5. Plasma fatty acids (*p* < 0.001) and cholesterol (*p* = 0.003) concentrations followed the same patterns and were greater in pigs fed HF diets compared to control; while the pigs fed CM had the greatest plasma fatty acid concentration. There was no effect of diet on plasma glucose, insulin, urea, IL-6, IL10, IGF-1, IGF-2, glucagon, GLP-1, TNFα, or C-peptide concentrations (Table 2). Besides IL10, which had a greater plasma concentration in week 2 compared to week 5 (139 vs. 72.6 pg/mL, SED 1.374, *p =* 0.075), there was no effect of week or the combination of diet and week on any of the plasma variables measured (data not shown).

### 3.2. IVGTT Responses

Unless otherwise specified, the results for camel milk are presented as the combination of raw and pasteurized milks as there was no statistical difference between each type.

**Figure 1:**
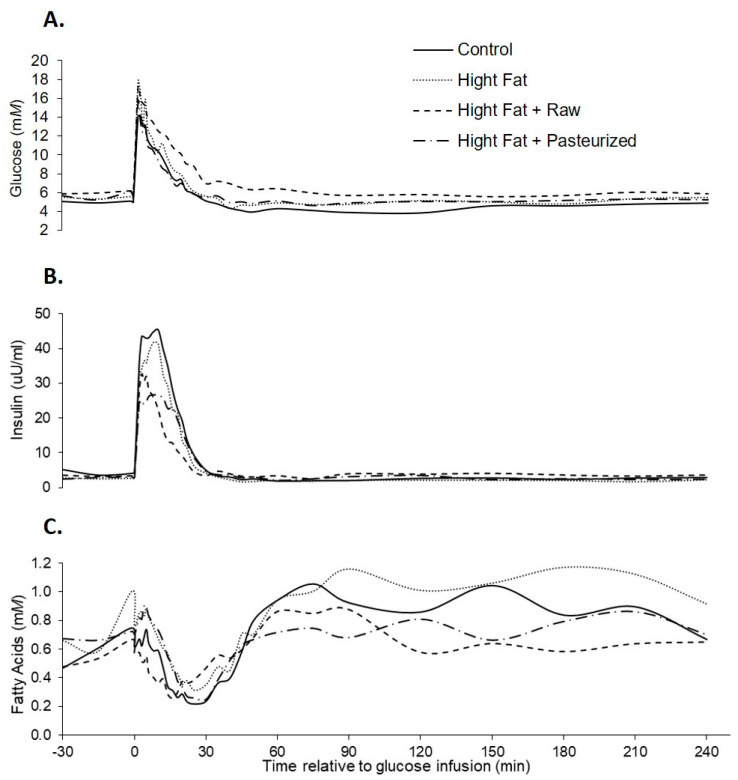
Plasma glucose (**A**), insulin (**B**), and fatty acids (**C**) responses to an in vitro glucose tolerance test (IVGTT) in female grower pigs (*n* = 20) fed either of the following: control, high fat, high fat + 500 mL/day pasteurized camel milk, or high fat + 500 mL/day raw camel milk. The glucose infusion occurred at time 0.

Comparable to the results obtained from the samples at weeks 2 and 5, basal plasma glucose, insulin, and fatty acids concentrations did not differ between pigs due to dietary treatment. Plasma glucose concentrations increased rapidly in response to the glucose infusion (Figure 1), reaching peak glucose, insulin, and fatty acids concentrations at 16, 8, and 118 min, respectively. Glucose concentrations returned to baseline in all treatment groups, indicating no presence of glucose intolerance.

The peak insulin concentration tended to be reduced in CM compared to control, although there was no difference between CM and HF treatments (Table 3). There was no effect of diet on peak plasma glucose and fatty acids concentrations, while peak plasma insulin concentrations tended to be (*p* = 0.058) lower in pigs fed a high fat and CM diet (Table 3). The glucose nadir obtained after the glucose infusion was less in pigs fed a control diet (*p* = 0.009), while the time taken to achieve this nadir did not differ with dietary treatment. There was no effect of diet on plasma glucose, insulin, or fatty acids concentrations at the end of the challenge period (240 min). The insulin AUC 0–30 tended to be greater for control compared to HF and CM treatments. The HOMA-IR index did not differ between dietary treatments (1.1 vs. 0.78 vs. 0.94 for control, HF, and CM, respectively, SED 0.377, *p* = 0.67). The insulin increment from basal tended to be greatest in control treatment and lowest in CM treatment (*p* = 0.055). The insulin decrement from basal was lower in HF and CM treatments compared to control (*p* = 0.028).

Minimal modelling results are presented in Table 4. There was no effect of CM on calculated parameters of insulin sensitivity or resistance. Pigs fed control diets had greater β-cell function compared to those fed CM and HF diets, while there was no difference between the CM and HF diets. The AIRg tended to be reduced by HF and CM diets, with the CM diet demonstrating the lowest AIRg.

## 4. Discussion

The combined results for all measures presented in the present experiment demonstrated no variance in responses between CM fed as pasteurized or raw. This is a positive finding as it suggests that the heat treatment of CM does not damage the active compounds and negate any beneficial impacts of CM consumption. As no differences were noted, the present experiment suggests that CM can be pasteurized without impacting insulinogenic properties.

To the best of our knowledge, this is the first experiment examining the potential impacts of CM on glycemic control in pigs, with all other published controlled experiments utilizing rodent models. By using the pig as a monogastric model, this experiment is a suitable proxy for human digestive systems, although the HF diet utilized here was unable to fully induce a Type 2 (insulin resistance) diabetic model, while the induction of Type 1 diabetes would have required drug interventions (such as streptozotocin). There are few published studies that have utilized minimal modelling to estimate indices of glucose and insulin dynamics in pigs. However, compared to previous work in pigs [17], the minimal modelling showed that the pigs fed the HF diet in this experiment had reduced insulin baseline levels but similar glucose baseline levels compared to those fed a control diet. While it was not expected to induce a full diabetic model, the HF diet was anticipated to induce a degree of insulin resistance and hence a greater fasting glucose concentration. There was no effect of HF diet consumption on basal plasma glucose, insulin, or urea concentrations, while circulating plasma fatty acid concentrations increased. Metabolic syndrome results in insulin resistance mainly noted in peripheral tissues (whole body glucose uptake) and is not noted in hepatic tissues (glucose production) and thus is not a true diabetic state. This lack of response to such a high fat diet (above 16% fat) is potentially driven by the improved genetics of the commercial strain of pig utilized, as these pigs are selected for their ability to rapidly grow and have increased drives for feed intake. In this experiment we were successful in numerically inducing a mild form of hyperglycemia (classified as circulating fasting glucose of 5.6–7.0 mmol/L); a true diabetic model would have fasting glucose concentrations greater than 8.0 mmol/L. Insulin resistance in pigs is directly related to body fat [18] but the failure to alter backfat thickness with the high fat diet is again indicative of the little effect on fat deposition and metabolic syndrome.

Although weekly blood samples did not demonstrate any alterations to insulin or glucose parameters, the responses to the IVGTT suggest that the HF diet was successful in stimulating a degree of insulin resistance in these pigs. The IVGTT responses demonstrated a lower insulin peak in response to glucose infusion in pigs fed the HF diet with the lowest in those fed CM. This was also reflected by a tendency for a lower AUC 0–30 min. This could suggest that there was more damage to β-cells, which were therefore not producing adequate insulin, but as there was no concurrent change in glucose maximum, this suggests that the lower insulin peak is adequately working to return to glucose homeostasis. Other studies show no change in fasting glucose but a reduction in insulin dose for diabetic humans consuming CM [3,4], which suggests that the effects of CM are not necessarily just as an insulin alternative (though it may be), but perhaps CM can also improve insulin effectiveness and/or uptake. Recent evidence shows that CM can inhibit the actions of dipeptidyl peptidase IV (DPP-IV) and increase the activation of insulin receptors [19]. This is supported by Abdulrahman et al. (2016) who demonstrated that CM directly enhances cellular glucose uptake via the improved activation and conformation of insulin receptors [18]. The experiment presented here further supported this interpretation as demonstrated by the lower insulin increment and decrement in response to the IVGTT in CM-fed pigs. A lack of change from the insulin baseline suggests tighter control of insulin secretion in response to glucose in CM-fed pigs, although this was not reflected by modelled changes in glucose mediated insulin responses.

In support of the findings presented in the present experiment, CM reduced circulating fasting glucose and increased insulin concentrations in diabetic but not control rats [20]. Similarly, in young humans (11–18 years) with confirmed metabolic syndrome (correlated with insulin resistance), 250 mL fermented CM/day for 8 weeks had numerical but non-significant effects on fasting blood glucose, inflammatory markers, serum free fatty acids, and incretin hormone concentrations, while there was a numerical (also non-significant) elevation of fasting insulin [21]. It was suggested that the lack of response was due to CM eliciting no hypoglycemic effects in subjects with near-normal glucose metabolisms [21]. This increase in insulin concentration contrasts with our results whereby there was no noted change in basal plasma insulin in pigs fed CM.

In diabetic humans, 500 mL daily CM consumption over one [22] and two years [3] reduced basal glucose concentrations but did not result in a change in basal insulin concentration, while CM reduced insulin requirements by 46%. In a similar shorter-term study, CM did not alter plasma insulin, C-peptide, or triglyceride concentrations, while low density lipoproteins were decreased after 3 months of CM consumption [23]. In the 3-month study, CM reduced glycosylated hemoglobin (HbA1c) concentrations [23], and while this occurred numerically in the longer term (2 year) CM study, this reduction was not significant [3]. As HbA1c is a marker of glycemic control, a reduction in this value demonstrates improved glycemic control. In our experiment, the pigs fed CM had a lower percentage change in glucose and insulin. This suggests (although additional research is required to confirm) that CM is improving glycemic control.

Minimal modelling output showed that pigs fed the HF diet had decreased β-cell function. These responses are indicative of the body’s ability to produce insulin in response to an increased glucose load. The reduced β-cell function predicted by the minimal modelling contrasts the suggestion of [17] that CM improves β-cell function [24]. However, this reduction in β-cell function is driven by two high values for the control group and therefore this result may be a manifestation of inherent differences between individual animals that might be eliminated with a larger sample size. Further, supporting results observed in diabetic humans [17], there was no significant impact of diet on circulating C-peptide concentrations in the present experiment. C-peptide is commonly measured as a marker of pancreatic β-cell function in humans and has a longer half-life than insulin, making it a useful circulating marker. Others [15] demonstrated only a small (non-significant) improvement in β-cell function in diabetic humans fed CM, even though the CM-fed group required less insulin to achieve better glycemic control than the comparison control group [22]. The authors suggest that this lack of change in β-cell function may be due to the timeframe (12 months) of the study being insufficient to elicit improvements in β-cell function [22], which is perhaps why such changes were not noted in the present experiment. As modelling demonstrated that CM did not result in changes to insulin secretion but potentially reduced β-cell function, it is possible that CM results in reductions in β-cell work leading to β-cell rest, or CM immunoglobulins may cause a reduction in immune system scavenging of β-cells, which overall increases insulin effectiveness without altering β-cell function.

Insulin not only acts on the enzymes involved in the intermediary metabolism of glucose, it also promotes glucose transport across the membrane of target cells. Insulin binds to the receptor in a complex manner with high affinity, which triggers signal transduction via multiple pathways that regulate the metabolic effects of insulin and regulate gene expression, cell growth, and differentiation [25]. In a cell culture model, CM increased the efficacy but not the potency (suggesting no impact on binding properties) of insulin and acted on downstream insulin signaling [26]. Conversely, glucagon acts to increase circulating glucose and fatty acid concentrations essentially functioning in opposing action to insulin. In the present experiment, there was no effect of diet on plasma insulin or glucagon concentrations. Insulin receptor signaling can also be attenuated by immune responses from cytokines such as interleukins (IL) and tumor necrosis factors (TNF). In Type 2 diabetic rats, CM attenuated the increased production of inflammatory markers (TNFα and TNFβ1), while no such change was noted in control CM fed rats [20]. In this experiment, there were no differences in plasma IL-6 (assists in the final differentiation of β-cells into immunoglobulin secreting cells), IL-10 (inhibits the synthesis of cytokines such as TNF), or TNFα concentrations. Further, CM can ameliorate hepatic damage caused by alcohol-induced hepatic injury in rats, which is also driven by the prevention of damage caused by oxidative stress responses as well as a significant reduction in liver triglycerides [27]. Taken together, these findings suggest that CM may attenuate immune induced suppression of insulin action. However, the lack of difference in plasma markers of inflammation observed in the present experiment do not support this hypothesis.

The incretin hormone GLP-1 acts to stimulate glucose-insulin secretion, inhibit glucagon secretion, and is involved in maintaining glucose homeostasis. Incretin hormones are produced in response to the presence of nutrients (such as fat and carbohydrates) in the digestive system, and in Type 2 diabetic (but not normal) rats a significant increase in GLP-1 can be attenuated by CM [20]. In our experiment, we did not observe any differences in plasma GLP-1 concentrations due to diet. Previous work showed that CM (plus honey) consumption decreased circulating glucose, increased insulin concentrations, and increased IGF-1 concentrations in type 1 diabetic rats [28]. IGF-1 has a functional role in the regulation of glucose and acts to increase peripheral tissue glucose absorption and suppress gluconeogenesis; it induces counter regulatory responses (both directly and indirectly) to suppresses pancreatic glucagon secretion. IGF-1 may have a role in the control of both insulin-dependent and non-insulin-independent diabetes due to its hypoglycemic actions and its ability to enhance insulin responsiveness during insulin resistant states [29]. IGF-2 has high structural homology with insulin and is produced in the liver and other tissues; symptoms similar to Type 2 diabetes (including hyperinsulinemia and mild hyperglycemia) are observed in transgenic mice that overexpress IGF-2 in β-cells [30] causing β-cell dysfunction that leads to diabetes [31]. However, in the present experiment no differences in circulating IGFs were observed, which suggests these signals are not involved in the modulatory actions of CM.

## 5. Conclusions

This experiment contributes towards the small existing body of work examining the potential positive benefits of CM on glycemic control. To our knowledge, this is the first experiment examining the effect of CM on glycemic control in pigs as a monogastric model. The combined results demonstrate that CM supplementation improved the efficacy of (or sensitivity to) insulin, likely by improving insulin effectiveness and/or uptake independent of changes to β-cell function (i.e., improved insulin sensitivity and decreased insulin resistance). The pigs fed CM also had a tighter control of insulin secretion in response to glucose, which occurred independent of the actions of inflammatory or incretin hormone responses. While these results contribute to the present understanding of the impact of CM on glucose homeostasis, further research is required to determine the impacts of CM on glycemic control and elucidate the mode of action. Specifically, further research using (drug induced) diabetic models in pigs are warranted.

## Figures and Tables

**Table 1:** Nutrient content of the control and high fat diets fed to growing pigs.

	Control	High Fat
DM (%)	90.7	91.6
DE (MJ/kg)	14.2	17.4
CP (%)	18.4	18.4
Fat (%)	2.7	16.2
Starch (%)	56.3	44.5
Fiber (%)	2.4	2.0

**Table 2:** Plasma glucose, fatty acids, and urea concentrations measured in growing female pigs (mean of two measurements at weeks 2 and 5 of a 6 week feeding period) fed either a control (*n* = 5), high fat (*n* = 5), or 500 mL per day camel milk (combination of raw and pasteurized, *n* = 10) diet. Values presented are back transformed.

	Control	High Fat	Camel Milk	SED	*p*-Value
Glucose (mM)	0.816	0.842	0.863	0.0242	0.15
	(6.55)	(6.95)	(7.30)	-	
Insulin (uU/mL)	0.680	0.697	0.681	0.1173	0.99
	(4.79)	(4.98)	(4.80)	-	
Fatty acids (mM)	2.068 ^a^	2.29 ^b^	2.33 ^c^	0.05958	<0.001
	(117)	(195)	(212)	-	
Urea (mM)	0.912	0.790	0.878	0.1324	0.68
	(8.17)	(6.17)	(7.56)	-	
Cholesterol Mm	0.0547 ^a^	0.161 ^b^	0.195 ^c^	0.03682	0.003
	(1.13)	(1.45)	(1.57)	-	
IL-10 pg/mL	2.09	1.92	2.00	0.1686	0.68
	(122)	(83.9)	(99.5)	-	
IGF1 ng/mL	1.41	1.40	1.43	0.02807	0.49
	(25.8)	(25.0)	(26.9)	-	
IGF-2 ng/mL	1.61	1.61	1.58	0.02878	0.44
	(41.1)	(40.8)	(38.4)	-	
IL-6 pg/mL	2.81	3.34	2.56	0.4377	0.19
	(640)	(2208)	(362)	-	
Glucagon pg/mL	2.39	2.21	2.52	0.1897	0.23
	(248)	(162)	(334)	-	
GLP1 ng/mL	1.27	1.25	1.14	0.07736	0.17
	(18.5)	(17.8)	(13.6)	-	
TNFa pg/mL	1.88	2.97	1.94	0.2822	0.93
	(75.9)	(923)	(87.7)	-	
C-Pep. ng/mL	0.441	0.616	0.593	0.09457	0.18
	(2.76)	(4.13)	(3.92)		

All data were analyzed after a logarithm transformation was performed and figures in parentheses are back-transformed means. ^abc^ Mean values in the same row with different superscript letters differ significantly (*p* < 0.05).

**Table 3:** Plasma glucose, insulin, and fatty acids parameters derived from intravenous glucose tolerance tests (IVGTT) including basal, peak, nadir and recovery concentrations, area under the curve (AUC), clearance rate (CR), increment and decrement from basal measured in growing female pigs fed either a control (*n* = 5), high fat (*n* = 5), or camel milk (combination of raw and pasteurized *n* = 10) diet. Values with different alphabetical superscripts differ statistically (*p* < 0.005).

		Control	High Fat	Camel Milk	SED	*p*-Value
Base (mM)	Glucose	5.03	5.51	5.81	0.603	0.28
	Insulin	4.94	3.09	3.46	1.199	0.22
	NEFA	0.649	0.723	0.663	0.1371	0.83
Peak (mM)	Glucose	14.7	17.7	16.6	2.579	0.46
	Insulin	57.9	44.6	33.4	12.04	0.058
	NEFA	1.32	1.39	1.13	0.1744	0.18
Nadir (mM)	Glucose	3.03 ^a^	4.22 ^b^	4.73 ^c^	0.606	0.009
	Insulin	1.69	1.22	2.28	1.137	0.38
	NEFA	0.177	0.256	0.248	0.0520	0.23
AUC 0–30 (mM.min)	Glucose	94	88	119	45.0	0.63
	Insulin	772	508	425	192.1	0.097
	NEFA	−7.3	−6.9	4.3	3.74	0.52
CR 2–30 (%/min)	Glucose	3.90	3.24	3.65	0.6860	0.61
	Insulin	7.25	7.05	6.67	1.806	0.91
	NEFA	3.75	3.33	2.48	1.859	0.65
Increment from basal (mM)	Glucose	9.66	12.2	10.8	2.315	0.54
	Insulin	52.9	41.5	30.0	11.23	0.055
	NEFA	0.668	0.671	0.470	0.1382	0.12
Decrement from basal (mM)	Glucose	2.00	1.30	1.09	0.5680	0.15
	Insulin	3.25 ^a^	1.87 ^b^	0.980 ^b^	0.9700	0.028
	NEFA	0.472	0.466	0.425	0.1310	0.88

^abc^ Mean values in the same row with different superscript letters differ significantly (*p* < 0.05).

**Table 4:** Minimal modelling (MINMOD) parameters derived from intravenous glucose tolerance tests (IVGTT) measured in growing female pigs fed either a control (*n* = 5), high fat (*n* = 5), or camel milk (combination of raw and pasteurized, *n* = 10) diet. AIRg = acute insulin response to glucose; DI = disposition index; GEZI = glucose effectiveness at zero insulin; G_0_ = distributed glucose concentration at time 0; SI = insulin sensitivity; SG = glucose effectiveness.

	Control	High Fat	Camel Milk	SED	*p*-Value
Insulin baseline (mU/L)	4.89	3.05	3.50	1.0050	0.22
Glucose baseline (mg/dL)	86.5	94.6	101	8.762	0.21
AIRg (mU.L^−1^.min)	372	274	223	62.80	0.059
β-cell function (mU/mM)	84.9 ^a^	35.7 ^b^	33.8 ^b^	15.48	0.006
DI	9312	5346	13,078	9915.0	0.72
GEZI (min^−1^)	0.023	0.027	0.041	0.0171	0.44
G_0_ (mg/dL)	252	271	295	29.38	0.29
Insulin Resistance (mM.mU/L^−2^)	1.03	0.745	0.916	0.2963	0.68
SI ((mU/L)^−1^.min^−1^)	30.7	18.4	65.9	53.21	0.60
SG (min^−1^)	0.0345	0.0329	0.0637	0.02415	0.29

^ab^ Mean values in the same row with different superscript letters differ significantly (*p* < 0.05).

## Data Availability

The data presented in this study are available from the corresponding author, upon reasonable request.
